# Analysis of drug combinations: current methodological landscape

**DOI:** 10.1002/prp2.149

**Published:** 2015-05-20

**Authors:** Julie Foucquier, Mickael Guedj

**Affiliations:** Department of Bioinformatics and Biostatistics, PharnextIssy-Les-Moulineaux, France

**Keywords:** Bliss, combination index, drug combination, loewe, synergy

## Abstract

Combination therapies exploit the chances for better efficacy, decreased toxicity, and reduced development of drug resistance and owing to these advantages, have become a standard for the treatment of several diseases and continue to represent a promising approach in indications of unmet medical need. In this context, studying the effects of a combination of drugs in order to provide evidence of a significant superiority compared to the single agents is of particular interest. Research in this field has resulted in a large number of papers and revealed several issues. Here, we propose an overview of the current methodological landscape concerning the study of combination effects. First, we aim to provide the minimal set of mathematical and pharmacological concepts necessary to understand the most commonly used approaches, divided into effect-based approaches and dose–effect-based approaches, and introduced in light of their respective practical advantages and limitations. Then, we discuss six main common methodological issues that scientists have to face at each step of the development of new combination therapies. In particular, in the absence of a reference methodology suitable for all biomedical situations, the analysis of drug combinations should benefit from a collective, appropriate, and rigorous application of the concepts and methods reviewed here.

## Introduction

Traditional and modern medicine has always taken advantage of the combined use of several active agents to treat different diseases. Based on a practice of more than 2000 years, traditional Chinese medicine uses mixtures of naturally occurring herbs (Yuan 2000). Since the last century, advances in Omics and Cell Biology have greatly impacted on the increasing use of drug combination in modern medicine (Keith et al. 2005). The enhanced understanding of the biology of a disease as a disturbed system of interconnected molecular pathways which are more susceptible to the simultaneous action of several drugs, provides new opportunities for the rational development of combination therapies (Smalley et al. 2006; Kitano 2007; Zimmermann et al. 2007; Podolsky and Greene 2011) and exploits the chances for better efficacy, decreased toxicity, and reduced development of drug resistance.

For these reasons, combination therapies have become a standard in several areas such as cancer (Humphrey et al. 2011), hypertension (Glass 2004), asthma (Nelson 2001), and AIDS (Larder et al. 1995; Oversteegen et al. 2007). In addition, for the pharmaceutical industry which is currently facing a decline in the discovery and approval of new molecular entities, reformulating existing drugs into combination products represents an essential strategy in indications of unmet medical need such as Alzheimer’s disease (Herrick and Million 2007; Pangalos et al. 2007).

For this purpose providing evidence of significant superiority of a combination of drugs compared to the single agents is of particular interest. Research in this field has resulted in a large number of theoretical and experimental papers (Greco et al. 1995; Chou 2006), and also revealed several methodological issues and caveats (Berenbaum 1977; Caudle and Williams 1993; Nieuwenhuis et al. 2011; Ocana et al. 2012; Geary 2013). In particular the concepts of *synergy* or *antagonism* have clear and well-accepted definitions: they represent, respectively, greater or lesser effects for drugs in combination than the simple *additive* effect expected from the knowledge of the effects of each drug individually. However, translating them into a valid methodology is a tricky problem that generally begins with the formal definition of additivity, and to which many but often not simple solutions have been proposed (Berenbaum 1977; Greco et al. 1995; Grabovsky and Tallarida 2004; Chou 2006; Geary 2013).

For 25 years, several authors have reviewed the subject, alerting about the incorrect use of terminologies and methodologies (Berenbaum 1977, 1989; Caudle and Williams 1993; Nieuwenhuis et al. 2011; Ocana et al. 2012; Berthoud 2013; Geary 2013), and stimulating the discussion on the appropriate approach to apply in practice (Berenbaum 1989; Greco et al. 1995; Chou 2006; Tallarida 2006; Geary 2013).

Here, we propose an overview of the current methodological landscape concerning the study of combination effects. First, we aim to provide the minimal set of mathematical and pharmacological concepts necessary to understand the most commonly used approaches, divided into *effect*-based approaches and *dose–effect*-based approaches, and introduced in light of their respective practical advantages and limitations. When possible, we provide a Combination Index (CI) recognized as the standard measure of combination effect that indicates a greater (CI < 1), lesser (CI > 1) or similar (CI = 1) effect than the expected additive effect. Then, we discuss the main common methodological issues that scientists have to face at each step of the development of new combination therapies. By way of illustration, we will consider the combination of two drugs *A* and *B*, administered at doses *a* and *b*, of respective effects *E*_*A*_ and *E*_*B*_, and of combined effect *E*_*AB*_. Main notations are defined in Data S1.

## Effect-Based Strategy

**Figure 1:**
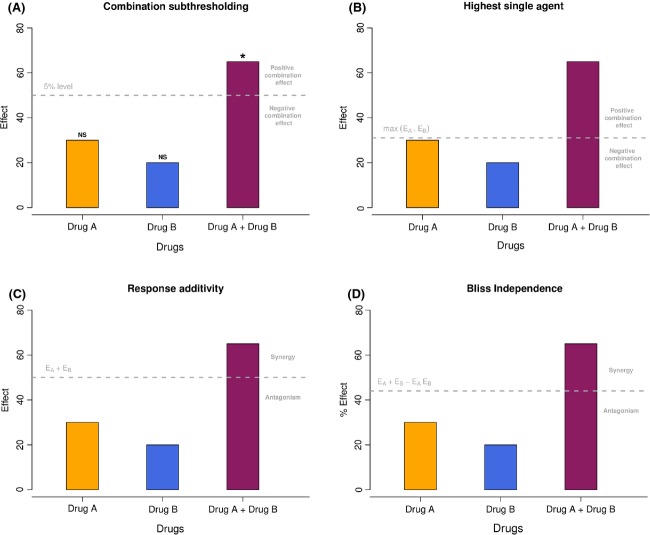
Illustration of the four effect-based approaches. (A) Combination Subthresholding, (B) Highest Single Agent, (C), Response Additivity, and (D) Bliss Independence. Based on *E*_*A*_ = 30, *E*_*B*_ = 20, and *E*_*AB*_ = 65. NS, Nonsignificant; *, Significant at the 5% level.

Methods following an effect-based strategy compare the effect resulting from the combination of two drugs (*E*_*AB*_) directly to the effects of its individual components (*E*_*A*_ and *E*_*B*_). The exact decision process that allows a conclusion of positive, negative, or null combination effect can vary among four main strategies which are (1) Combination Subthresholding, (2) Highest Single Agent, (3) Response Additivity, and (4) Bliss Independence model described hereafter and illustrated in Figure1.

The *Combination Subthresholding* approach consists in showing that combination of noneffective doses of drugs yields significant effect (Fig.1A). Effectiveness is generally declared based on a *P*-value resulting from a statistical test versus an untreated control group below the 0.05 level. The application of this simple approach is common. However, effectiveness declared based on a threshold does not necessarily imply a convincing difference between the effect of the drug combination and the effects of its individual components (Nieuwenhuis et al. 2011). Consider an extreme scenario in which the drug combination barely reaches significance (e.g., *P* = 0.049) and its individual components narrowly failed to reach significance (e.g., *P* = 0.051). Although these effects lie on opposite sides of 0.05, the difference between “significant” and “not significant” is not itself necessarily significant, and one cannot be convinced of a positive improvement in effect by the drug combination compared to the drugs taken individually.

The *Highest Single Agent* approach (Lehár et al. 2007) (also referred to as the Gaddum’s noninteraction (Berenbaum 1989) or cooperative effect (Geary 2013)) simply reflects the fact that the resulting effect of a drug combination (*E*_*AB*_) is greater than the effects produced by its individual components (*E*_*A*_ and *E*_*B*_) (Fig.1B). A Combination Index can be calculated as: 
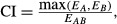
 and the significance of a positive effect is given by the *P*-value of the statistical test comparing the combination to the highest single agent. The *Highest Single Agent* approach represents an improvement compared to the previous *Combination Subthresholding* approach, interpreting the significance of differences rather than the difference of significances. It provides evidence of the superiority of the drug combination compared to its single agents. However, by comparing the combination directly to the highest single agent, this approach fails to demonstrate an improved drug combination effect compared to the expected additive effect of its individual components. As a consequence, a positive result obtained with the *Highest Single Agent* approach indicates a positive drug combination effect with an amelioration compared to the single drugs considered alone, but provides a very limited evidence of synergy except in the case where at least one drug is known to be inactive at any concentration.

**Figure 2:**
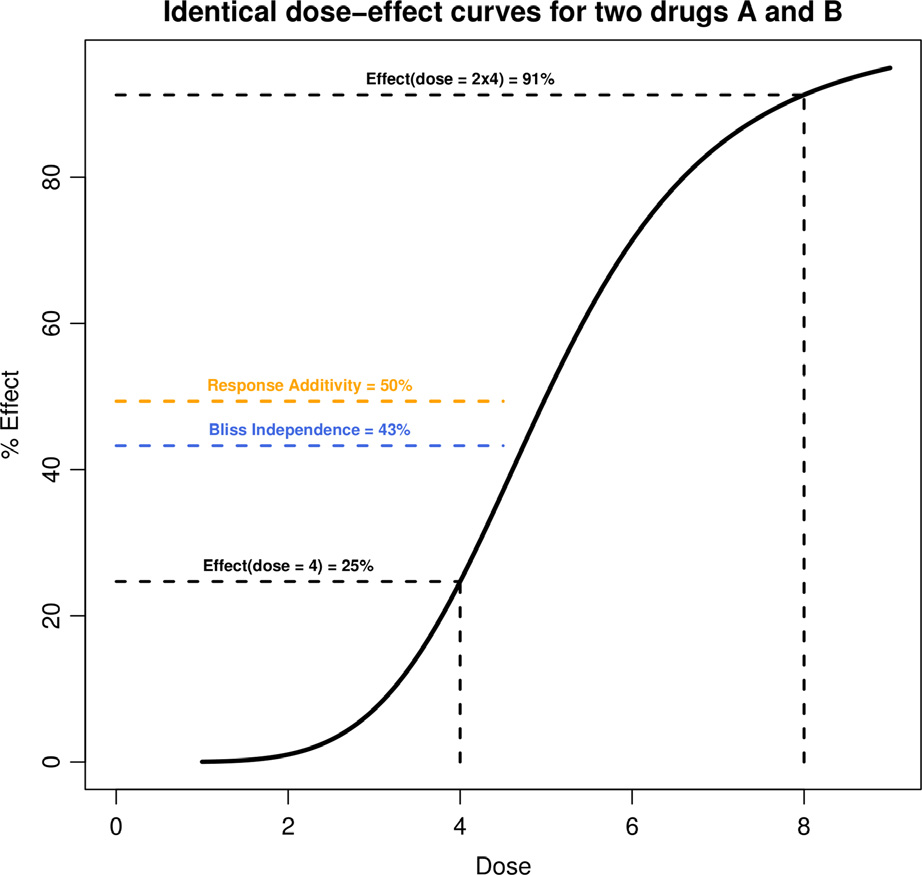
Possible inconsistency in assessing drug synergy based on Response Additivity or Bliss Independence. Identical simulated dose–effect curve for two different drugs. Suppose that a dose = 4 of drug *A* results in 25% of effect, likewise for drug B. From *Response Additivity*, one would conclude in synergism with a combination effect above 50%. From *Bliss Independence*, one would conclude in synergism with a combination effect above 43%. However, note that either a dose = 2 × 4 = 8 of drug *A* or of drug *B* alone brings the effect up to 91%. Therefore, a total of dose = 8 of the hypothetical combined drug elicits less effect under *Response Additivity* or *Bliss Independence* than the same dose of either drug alone, yet one would conclude synergism.

The *Response Additivity* approach (also referred to as Linear Interaction Effect (Slinker 1998)) consists in showing that a positive drug combination effect occurs when the observed combination effect (*E*_*AB*_) is greater than the expected additive effect given by the sum of the individual effects (*E*_*A*_* *+ *E*_*B*_) (Fig.1C). The Combination Index can be calculated as: 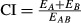, and the corresponding *P*-value is given by the significance of the interaction effect in a factorial analysis of variance of the individual and combination effects (Slinker 1998). The *Response Additivity* approach can appear as a natural improvement of the *Highest Single Agent* to assess synergy, as it compares the observed combination effect (*E*_*AB*_) to an expected effect from additivity instead of the effect of the single agents. It assumes that drugs have linear dose–effect curves with zero intercepts, which is generally not the case as most dose–effect curves are characterized by logistic or curvilinear shapes (Caudle and Williams 1993). To better understand the problem, Figure2 illustrates the simple and extreme case in which two dose–effect curves are identical and the combination effect is merely additive. *Response Additivity* would indicate synergism in the curved-up part and antagonism in the curved-down part, and would result in the following invalid and counterintuitive interpretation of the drug combination effect: a synergistic combination could be less effective than its components applied individually. Figure2 can also be used to illustrate the paradox of the sham combination of one drug with itself. Let’s say that a drug preparation is divided into two tubes, and then each tube is treated as if it contained a different drug with identical dose–effect curves. By using the same logic, one could conclude that the combination of the same drug with itself (obviously additive) is synergistic (Greco et al. 1995).

The *Bliss Independence* model (Bliss 1939; Berenbaum 1989; Greco et al. 1995; Geary 2013) is based on the principle that drug effects are outcomes of probabilistic processes and assumes that drugs act independently in such a manner that neither of them interferes with the other (different sites of action), but each contributes to a common result (Fig.1D). The observed combination effect expressed as a probability (0 ≤ *E*_*AB*_* *≤ 1) can be compared to the expected additive effect given by the common formula for probabilistic independence *E*_*A*_ + *E*_*B*_(1 − *E*_*A*_) = *E*_*A*_ + *E*_*B*_ − *E*_*A*_*E*_*B*_, where 0 ≤ *E*_*A*_ ≤ 1 and 0 ≤ *E*_*B*_ ≤ 1. The resulting Combination Index can be calculated as: 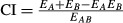. The *Bliss Independence* model is considered as one of the most popular models to assess the combined effects of drugs, but it presents some limitations (Goldoni and Johansson 2007). First, the search for synergy often involves drugs with multiple, complex, possibly unknown mechanisms of action, and therefore, methodologies should not depend upon knowledge of mechanisms of action (Greco et al. 1995). Then, *Bliss Independence* assumes that the drugs have exponential dose–effect curves (Berenbaum 1989), which could lead the same counterintuitive interpretation discussed for *Response Additivity* and illustrated in Figure2. Finally a main limitation is that the model applies only to effects expressed as probabilities ranging within 0 and 1.

## Dose–Effect-Based Strategy

Opponents to the effect-based approaches consider that the proper way to compare different agents having nonlinear dose–effect curves is to find what amount or concentration of each produces the same quantitative effect, which can be referred to as dose–effect-based approaches (Berenbaum 1977). The expected (additive) effect of a combination depends on the individual dose–effect curves and enables the formulation of unequivocal definitions of synergy, additivism, and antagonism. In particular dose–effect-based approaches rely on the mathematical framework known as *Loewe Additivity*, since it was first mentioned by Frei (1913) (Frei 1913) but first defined formally by Loewe (1926) (Loewe 1926, 1927, 1953, 1959).

### Mathematical framework

**Figure 3:**
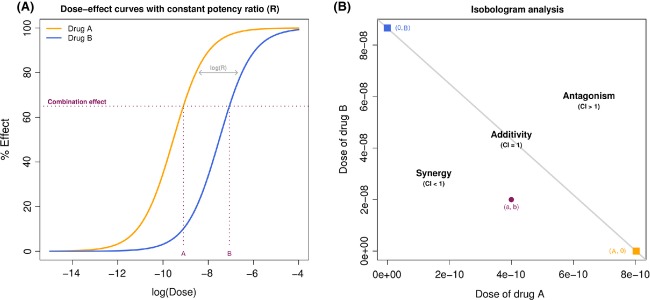
Illustration of the Loewe Additivity. (A) Dose–effect curves for two drugs *A* and *B* (here with constant potency ratio *R*) allow estimation of the single doses *A*_*E*_ and *B*_*E*_ reaching the combination effect *E* produced by the combination of doses *a* of drug *A* and *b* of drug *B*. (B) Isobologram analysis at the combination effect *E*. The single doses *A*_*E*_ and *B*_*E*_ are used to draw the line of additivity. The localization of the experimental point (*a*, *b*) corresponding to the doses actually needed for a combination effect *E* with respect to the line of additivity can be translated in term of synergy, additivity, and antagonism.

*Loewe Additivity* rests on both the *dose equivalence principle* (that for a given effect, dose *a* of drug *A* is equivalent to dose *b*_*a*_ of drug *B*, and reciprocally) and the *sham combination principle* (that *b*_*a*_ can be added to any other dose *b* of drug *B* to give the additive effect of the combination). The additive effect of drugs *A* and *B* depends on the individual dose–effect curves and can be expressed as:

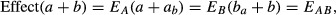
where *E*_*A*_ is measured on the dose–effect curve of drug *A*, (*a* + *a*_*b*_) corresponds to the dose *A* giving the effect *E*_*AB*_ and, respectively, for drug *B*. It makes the assumption that the drugs have a *constant potency ratio* (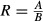). In practice, dose–effect curves with constant potency ratio have a constant ratio of doses at every level of effect and hence are parallel on a log-dose scale, and have equal individual drug maximum effects (Fig.3A) (Tallarida 2012). From there, we can easily define the following relation between all pairs of doses (*a*, *b*) producing the combination effect *E*_*AB*_ and the single doses *A* and *B* necessary to reach this effect:

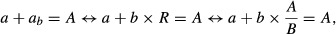
which leads to the most influential mathematical relation of the *Loewe Additivity* at the basis of most dose–effect-based approaches developed subsequently:

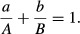


### Combination index

This relation first allows us to assess a Combination Index for the *Loewe Additivity* (Berenbaum 1977; Chou and Talalay 1983, 1984): 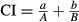. In practice a CI < 1 indicates that the doses *a* and *b* producing a given effect in combination are lower than the expected doses from additivity and can hence be directly interpreted as synergy. Similarly, a CI > 1 indicates that the doses *a* and *b* producing a given effect in combination are superior to the expected doses from additivity and can hence be directly interpreted as antagonism.

### Isobologram analysis

Another advantage of the *Loewe Additivity* is that it also enables us to complement the algebraic analysis with an intuitive, flexible and widely accepted graphical approach known as isobologram analysis (Greco et al. 1995). Given an effect *E* produced by the combination of doses *a* of drug *A* and *b* of drug *B*, the equation 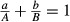 defines all the pairs of doses of drugs *A* and *B* that should lead to the combination effect *E*_*AB*_ from additivity, and can be drawn as a line of additivity of negative slope (
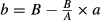
, also called *additive isobole*) on a graph where the *x* and *y* axes represent the dose of drugs *A* and *B* (Fig.3B). This representation makes clear that when drug *A* is present at dose *A* the quantity of drug *B* needed to reach the specified level is zero, and that the presence of drug *B* reduces the need for drug *A* in a quantity predicted by the model. Then the localization of the experimental point (*a*, *b*) corresponding to the doses actually needed for a combination effect *E*_*AB*_ with respect to the line of additivity can be translated in term of synergy, additivity and antagonism (Grabovsky and Tallarida 2004; Chou 2006; Geary 2013): an experimental point below the line corresponds to a CI < 1 and indicates synergy; a point on the line corresponds to a CI = 1 and indicates simple additivity; finally a point above the line corresponds to a CI > 1 and indicates antagonism (Fig.3B).

### Practical limitations

We have identified two main practical limitations of the combination analysis based on *Loewe Additivity*. First it relies on *accurately estimated dose–effect curves* to support the calculation of the effective doses (*A* and *B*) for a given effect (*E*_*AB*_). In most cases, the dose–effect relationship follows the Hill equation (also called sigmoid or logistic function) defined by:

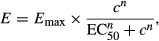
where *E* is the effect reached at concentration *c*, *E*_max_ is the maximum effect, EC_50_ is the half maximum effective concentration and corresponds to the inflection point of the curve, and *n* is the shape parameter linked to the steepness of the curve. Estimation of dose–effect curves for the drugs being combined requires a certain amount of data and can rapidly become expensive as well as experimentally and computationally demanding, and makes the analysis of drug combination prohibitive (Lehár et al. 2007). *Loewe Additivity* model becomes unusable when a dose–effect curve is not available or difficult to model (Zhao et al. 2014).

Then, in practice, only in a limited number of situations are additive isoboles straight lines. The potency ratio (*R*) is often not constant, a situation that would apply when the individual log-dose–effect curves are not parallel and/or when the individual drug maximum effects differ and lead to *curvilinear additive isoboles* (Grabovsky and Tallarida 2004). The calculation of the Combination Index and the isobologram analyses in such situations although more technical, are feasible as detailed in Data S2 following the work of Grabovsky and Tallarida (2004) (Grabovsky and Tallarida 2004).

Finally a number of other algebraic and graphical approaches have been built based on *Loewe* equations. Most are reviewed and discussed in Greco et al. (1995) (Greco et al. 1995). It is at least worth mentioning the median-effect approach of Chou and Talalay analyzing combination effects on the basis of the principle of mass action, and which has been the subject of a number of publications (Chou and Talalay 1983, 1984; Chou 2006, 2010).

## Current Issues in the Analysis of Drug Combinations

We have identified six main issues that we address here and that should be further considered in future developments and publications.

### Issue 1: the analysis of drug combinations requires an appropriate use of concepts and methods

The term “synergy” is used extensively as a gold standard to justify drug combinations when designing clinical studies. However, it has been shown that the literature is often obscure and is profusely littered with technical terms that are not always clearly defined (Berenbaum 1977), and that in most studies, the term “synergy” is used without appropriate understanding of either the underlying concept or the methods necessary to evaluate it (Ocana et al. 2012; Berthoud 2013). It is clear from the paradoxes illustrated in Figure2 that the interpretation of drug combination effects requires a minimal knowledge of the single dose–effect curves within the range of effect of interest (Berenbaum 1977; Greco et al. 1995; Tallarida 2001; Goldoni and Johansson 2007; Lee and Kong 2009; Ocana et al. 2012). Experiments are commonly designed in such a way that they could not detect synergy even if it was present and results are interpreted as showing synergy when there is no evidence for it or as showing additivity where there is clear antagonism, and so on (Berenbaum 1977). Experiments in which any drug is tested at less than three dose levels are therefore not likely to be sufficient to demonstrate synergy (Berenbaum 1977). It is also worthy of mention that the positive combination effect of two drugs can take other forms than synergy. When only one drug is active alone, a greater combination effect is generally referred to as “*potentiation*.” When the drugs combined are not active alone an effective combination is termed “*coalism*.” A combination can also have an effect on a range of biological systems or anatomical sites that are not completely covered by any drug individually, a situation described as “*cooperation*” (Gordon Steel and Peckham 1979).

### Issue 2: the analysis of drug combinations requires a standard reference analysis framework

This framework should ideally (a) provide a clear definition of additivity, synergy, and antagonism, (b) not rely on knowledge of mechanisms of action that are often unknown or not well understood even for many common drugs such as aspirin (Jia et al. 2009), (c) be general enough to cover rare and specific cases, (d) not result in counterintuitive results, (e) be adapted to possible practical and ethical issues to obtain data, (f) be intuitive and user friendly to be adopted by most scientists. There is to date no universal method that fulfills all the aspects of this task and although useful, “all models are wrong” (Box and Draper 1987; Shafer 2012). Dose–effect approaches based on *Loewe Additivity* can best survive criticism (Greco et al. 1995), but the relatively large amount of data required can make combination experiments prohibitive when data are expensive or difficult to obtain. Effect-based approaches such as *Highest Single Agent*, *Response Additivity,* and *Bliss Independence* appear more adapted to practical limitations as one minimally needs three or four experimental points for their application: (0, 0), (*a*, 0), (0, *b*), and (*a*, *b*). Despite their limitations, they can provide sufficient and convincing evidence of positive combination effect. In this context, we feel that the search for a reference analysis framework will not find its solution in one ideal model but rather in using a set of appropriate methods adapted to each step of the research and development process from discovery to the marketing authorization application, as discussed in the next issue.

### Issue 3: the analysis of drug combination must be adapted to each step of the research and development process

The *discovery step* is often dedicated to the in vitro screening of combinations of a set of candidate drugs administered at various doses in order to identify one or several combinations of interest. For each drug, screening experiments should explore drug doses that span the anticipated region of activity upon and below the EC_50_ depending on the current state of knowledge. The combined application of methods such as the *Highest Single Agent*, *Bliss Independence,* and *Loewe Additivity* may be useful to identify good candidates for further mechanistic or clinical research (Borisy et al. 2003; Zhao et al. 2004, 2010; Lehár et al. 2007, 2009; Cokol et al. 2011).

Then in vitro and in vivo *preclinical studies* should determine more precisely the nature and extent of combination effects selected from the discovery step. At this stage, single dose–effect curves should be well characterized and a dose–effect approach based on *Loewe Additivity* with Combination Index and Isobologram analysis appears as the more suitable. In situations where dose–effect curves are not available for all the drugs combined such as *potentiation* or coalism, the *Highest Single Agent* approach is appropriate.

When moving to *clinical studies* in humans, the analysis of drug combinations has to face strong practical and ethical limitations, and it is generally nearly impossible to obtain sufficient data to clearly and properly support synergy. In this context, recommendations from the US Food and Drug Administration (FDA) [*Codevelopment of Two or More New Investigational Drugs for Use in Combination, 2013]*, the European Medicines Agency (EMA) [*Guideline on fixed combination medicinal products, 2008*] and the World Health Organization (WHO) [*Guidelines for registration of fixed-dose combination medicinal products, 2005*] have evolved markedly during the last decades (Podolsky and Greene 2011; Woodcock et al. 2011). In summary, the agencies agree that there should be compelling basis and rationale to justify the use of a combination therapy supported by the biology of the disease of interest and preclinical studies (preferably in animals), as well as strong reasons to justify that the components cannot be developed as individual agents (Woodcock et al. 2011) for the studied disease. The extrapolation of results from in vitro to animals, or from animals to humans is a general and separate scientific debate which is not expected to be solved here (Shanks et al. 2009; Chou 2010; Tsilidis et al. 2013; Hay et al. 2014). Then the clinical development should provide evidence that the combination has a greater efficacy than any of the active drugs given alone at the same dose, or results in a level of efficacy similar to the one achievable by each active drug at higher doses, with a better safety profile. For each phase, the amount and types of clinical data needed and appropriate study designs vary depending on the nature of the combination being developed, the disease to be treated and what is known from the previous phases. Often, a large four-arm clinical trial (placebo or standard of care, drug A, drug B, and combination) is needed to meet the requirement of the agencies (Woodcock et al. 2011). When the contribution of each drug to the combination is convincingly demonstrated from the previous phases, when it is not ethical to treat patients with a placebo or a suboptimal therapy of doses, or when the recruitment may be difficult or slow such as in rare indications, the required combination study design may be simplified.

### Issue 4: optimizing dose ratio

**Figure 4:**
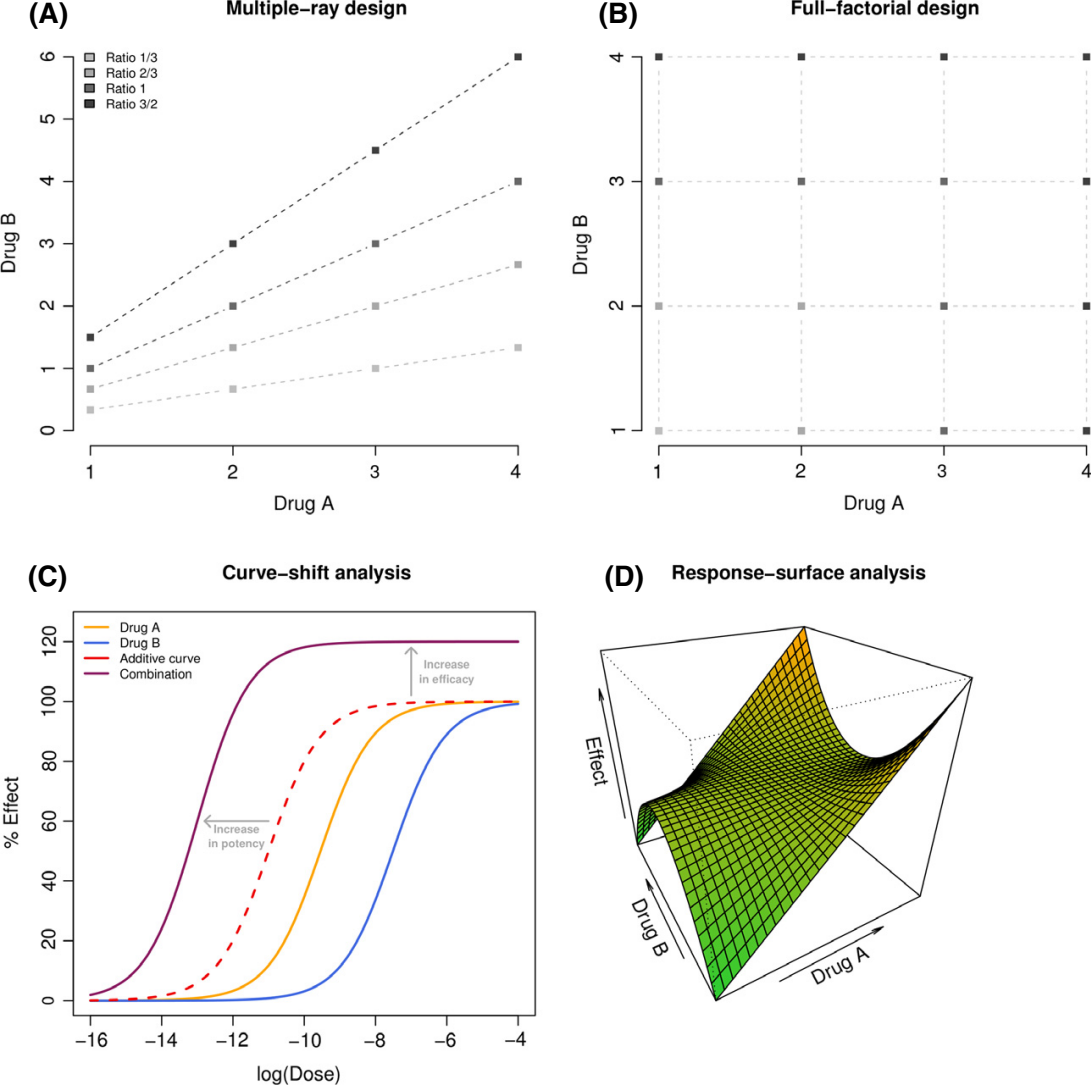
Optimizing dose ratio. (A) Multiple-ray design exploring 16 combinations (4 ratios × 4 doses). (B) *Full factorial design* exploring 16 combinations (4 × 4 doses). (C) *Curve-shift analysis*. The dose–effect curve for a combination at a given ratio (in purple) is compared to the additive expectation (in red) which can illustrate synergy by both an increase in potency and/or an increase in efficacy relatively to the single agent responses. Additive and combination curves are represented as functions of the dose of the more potent drug (here drug A). (D) *Response-surface analysis* can provide a complete description of the combination effect over a large range of doses.

The benefit of a combination therapy is not simply due to the property of the drugs, but could also depend on the dose ratio. As the cells do not make the difference between a single drug or a combination, two drugs combined at a given ratio could be considered as a third agent with its own dose–effect relation (Chou 2010). Therefore, rather than simply asking whether a particular combination is synergistic, we might do better to consider what dose ratio optimizes the synergy (Keith et al. 2005). For this purpose, a multiple-ray design (Fig.4A) exploring a given set of fixed ratios (the dose of one drug is escalated while the dose of the second remains constant) should be preferred to the full factorial design (Fig.4B) considering all the combinations of the selected doses of the individual drugs (Chou and Talalay 1984; Greco et al. 1995; Straetemans et al. 2005). From there different dose ratios can be compared by the mean of their respective dose–effect curves by applying a curve-shift analysis (Fig.4C) (Zhao et al. 2010), and a 3D response-surface analysis spanning the explored region of doses can provide a more complete description of the combination effect (Fig.4D) (Prichard and Shipman 1990; Greco et al. 1995; Breitinger 2012; Geary 2013). Ideally, the dose ratio should be optimized in preclinical studies before proceeding to clinical testing in humans.

### Issue 5: the interpretation of drug combination effects will benefit from more rigorous methodology

An additional problem in interpreting drug combination effects is the quality of the measured data: biological systems invariably carry experimental error, and thus borderline cases are almost impossible to assign (Breitinger 2012). For example, should we report a Combination Index of 0.97 as a true and convincing combination effect or as a simple deviation from 1 due to experimental variability? If the 95% confidence interval is of [0.95–0.99], we can conclude that the Combination Index is different from 1, whereas if it is of [0.85–1.05], the drug combination cannot be considered to show any effect deviating from the additivity, statistically speaking (Lee and Kong 2011). The *Highest Single Agent* and *Response Additivity* approaches come with a natural measure of significance as they are based on the statistical testing of the combination effect against the maximum individual effect. But Combination Indexes resulting from *Bliss Independence* and *Loewe Additivity* are generally reported without any assessment of the degree of certainty to be made from an experiment. These approaches lack the theoretical framework to allow for a direct statistical inference. Few papers have the merit to address this technical question and even these provide no simple answer to be implemented readily (Carter et al. 1988; Gennings et al. 1990; Belen’kii and Schinazi 1994; Lee and Kong 2009; Zhao et al. 2014). As methods can be complex and continue to evolve, statisticians and methodologists should be involved in all stages of drug combination research and development, which in practice is too often sporadically the case leading to flawed designs and analysis (Ioannidis et al. 2014). The development of standard softwares or libraries providing ready access to most of the methods described here is crucial and will also contribute in improving the quality and reproducibility of results. Only a few examples exist, focusing on one specific approach: the MixLow R package (http://cran.r-project.org/web/packages/mixlow) proposes an implementation of the *Loewe Additivity* (Boik and Narasimhan 2010); CompuSyn (http://www.combosyn.com) and CalcuSyn (http://www.biosoft.com/w/calcusyn.htm) implements the Median-Effect approach of Chou and Talalay (Chou and Talalay 1983, 1984; Chou 2006, 2010). Publications often mention the use of SAS (Wang et al. 1997) or R scripts (Zhao et al. 2010; Whitehead et al. 2013) but do not mention how methods are implemented (Raffa et al. 2000; Borisy et al. 2003; Lehár et al. 2007, 2009; Cokol et al. 2011).

### Issue 6: combining more than two drugs

Drug combination analysis is often presented on drug pairs in order to ease its understanding and because it covers the most common situation in practice. But combining more than two drugs is not so rare (in cancers chemotherapy regiments can easily reach four or five agents) and most of the methods described here can easily be extended for use with any number of drugs (Bliss 1939; Berenbaum 1978; Chou and Talalay 1983). For instance with more than two drugs combined, the Combination Index is generalized to:

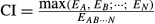
 for the *Highest Single Agent* approach,

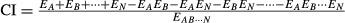
 for the *Bliss Independence* model,

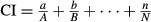
 for the *Loewe Additivity*. Note that when the combination counts three drugs, the equation CI = 1 corresponds to the plane passing through *A*, *B*, and *C* when doses of drugs *A*, *B*, and *C* are, respectively, presented by three coordinate axes, instead of a line when combining two drugs.

However, such a generalization to the analysis of more than two drugs does not allow investigation of the contribution of each drug to the whole combination effect. A combination of three drugs (A, B, and C) with a synergistic effect (CI < 1) could result from the synergistic effect between *A* and *B* only. A complete understanding of the contribution of each drug to the whole combination effect would require an assessment over all the subcombinations which is generally not feasible in practice. When the combination is based on logic such as the combination of two new investigational drugs (*A* and *B*) with a reference treatment (*C*), the analysis should follow the same logic in order to show here that *A* and *B* are synergistic, and that the combination of *A* + *B* (considered as a new single agent) with the reference treatment *C* is also synergistic.

## Conclusions and Prospects

Drug combination effects have been studied and analyzed by scientists for over 100 years. The advantages of combining drugs are well recognized, and activity in the area has increased dramatically thanks to the opportunities provided by the enhanced understanding of Systems Biology of disease (Keith et al. 2005; Zimmermann et al. 2007). This research has resulted in an immense number of theoretical and experimental papers in nearly all biological and medical sciences, and has involved scientists from many disciplines (Pharmacology, Mathematics, Epidemiology, and others) (Greco et al. 1995). Methods to generate and analyze data have evolved substantially over time thanks to substantial improvements in screening technologies and computational capacities (Cokol et al. 2011), but the main methodological issues remain appreciably the same (Prichard and Shipman 1990). As a significant part of the theoretical and empirical literature propagates fundamental misunderstandings (Berenbaum 1977, 1989; Caudle and Williams 1993; Nieuwenhuis et al. 2011; Ocana et al. 2012; Berthoud 2013; Geary 2013), future developments should benefit from a more appropriate and rigorous application of the concepts and methods discussed here for a proper assessment and interpretation of combination effects. In addition, in the absence of a reference methodology appropriate for all biomedical situations, the analysis of drug combinations will be facilitated by the collective use of different approaches. Finally, and beside the study design and analysis methodological aspects discussed here, research and pharmaceutical companies working on new combination therapies will have to face additional challenges such as elucidating the mechanisms of action by which drugs cause their single and joint actions (Jia et al. 2009) and improving the science behind formulating combination drugs.

## Supporting Information

**Data S1.** Notations. **Data S2.** Dose-effect based strategy: cases when dose-effect curves are not parallel.

## References

[b1] Belen’kii MS, Schinazi RF (1994). Multiple drug effect analysis with confidence interval. Antiviral Res.

[b2] Berenbaum M (1977). Synergy, additivism and antagonism in immunosuppression - critical review. Clin Exp Immunol.

[b3] Berenbaum MC (1978). A method for testing for synergy with any number of agents. J Infect Dis.

[b4] Berenbaum M (1989). What is synergy?. Pharmacol Rev.

[b5] Berthoud H-R (2013). Synergy: a concept in search of a definition. Endocrinology.

[b6] Bliss CI (1939). The toxicity of poisons applied jointly. Ann Appl Biol.

[b7] Boik JC, Narasimhan B (2010). An R package for assessing drug synergism/antagonism. J Stat Softw.

[b8] Borisy AA, Elliott PJ, Hurst NW, Lee MS, Lehar J, Price ER (2003). Systematic discovery of multicomponent therapeutics. Proc Natl Acad Sci USA.

[b9] Box GEP, Draper NR (1987). Empirical Model-Building and Response Surfaces.

[b10] Breitinger H-G, Acree W (2012). Drug synergy - mechanisms and methods of analysis. Toxicity and Drug Testing.

[b11] Carter W, Gennings C, Staniswalis J, Campbell E, White K (1988). A statistical approach to the construction and analysis of Isobolograms. Int J Toxicol.

[b12] Caudle RM, Williams GM (1993). The misuse of analysis of variance to detect synergy in combination drug studies. Pain.

[b13] Chou T-C (2006). Theoretical basis, experimental design, and computerized simulation of synergism and antagonism in drug combination studies. Pharmacol Rev.

[b14] Chou T-C (2010). Drug combination studies and their synergy quantification using the Chou-Talalay method. Cancer Res.

[b15] Chou T-C, Talalay P (1983). Analysis of combined drug effects: a new look at a very old problem. Trends Pharmacol Sci.

[b16] Chou TC, Talalay P (1984). Quantitative analysis of dose-effect relationships: the combined effects of multiple drugs or enzyme inhibitors. Adv Enzyme Regul.

[b17] Cokol M, Chua HN, Tasan M, Mutlu B, Weinstein ZB, Suzuki Y (2011). Systematic exploration of synergistic drug pairs. Mol Syst Biol.

[b18] Frei W (1913). Versuche Uber Kombination Von Desinfektionsmitteln.

[b19] Geary N (2013). Understanding synergy. Am J Physiol Metab.

[b20] Gennings C, Carter WJ, Campbell E, Staniswalis J, Martin T, Martin B (1990). Isobolographic characterization of drug interactions incorporating biological variability. J Pharmacol Exp Ther.

[b21] Glass G (2004). Cardiovascular combinations. Nat Rev Drug Discov.

[b22] Goldoni M, Johansson C (2007). A mathematical approach to study combined effects of toxicants in vitro: evaluation of the Bliss independence criterion and the Loewe additivity model. Toxicol In Vitro.

[b23] Gordon Steel G, Peckham MJ (1979). Exploitable mechanisms in combined radiotherapy-chemotherapy: the concept of additivity. Int J Radiat Oncol.

[b24] Grabovsky Y, Tallarida RJ (2004). Isobolographic analysis for combinations of a full and partial agonist: curved isoboles. J Pharmacol Exp Ther.

[b25] Greco WR, Bravo G, Parsons JC (1995). The search for synergy: a critical review from a response surface perspective. Pharmacol Rev.

[b26] Hay M, Thomas DW, Craighead JL, Economides C, Rosenthal J (2014). Clinical development success rates for investigational drugs. Nat Biotechnol.

[b27] Herrick TM, Million RP (2007). Tapping the potential of fixed-dose combinations. Nat Rev Drug Discov.

[b28] Humphrey RW, Brockway-Lunardi LM, Bonk DT, Dohoney KM, Doroshow JH, Meech SJ (2011). Opportunities and challenges in the development of experimental drug combinations for cancer. J Natl Cancer Inst.

[b29] Ioannidis JPA, Greenland S, Hlatky MA, Khoury MJ, Macleod MR, Moher D (2014). Increasing value and reducing waste in research design, conduct, and analysis. Lancet.

[b30] Jia J, Zhu F, Ma X, Cao Z, Cao ZW, Li Y (2009). Mechanisms of drug combinations: interaction and network perspectives. Nat Rev Drug Discov.

[b31] Keith CT, Borisy AA, Stockwell BR (2005). Multicomponent therapeutics for networked systems. Nat Rev Drug Discov.

[b32] Kitano H (2007). A robustness-based approach to systems-oriented drug design. Nat Rev Drug Discov.

[b33] Larder BA, Kemp SD, Harrigan PR (1995). Potential mechanism for sustained antiretroviral efficacy of AZT-3TC combination therapy. Science.

[b34] Lee JJ, Kong M (2009). Confidence intervals of interaction index for assessing multiple drug interaction. Stat Biopharm Res.

[b35] Lee JJ, Kong M (2011). Rebuttal to the response of Chou. Cancer Res.

[b36] Lehár J, Zimmermann GR, Krueger AS, Molnar RA, Ledell JT, Heilbut AM (2007). Chemical combination effects predict connectivity in biological systems. Mol Syst Biol.

[b37] Lehár J, Krueger AS, Avery W, Heilbut AM, Johansen LM, Price ER (2009). Synergistic drug combinations tend to improve therapeutically relevant selectivity. Nat Biotechnol.

[b38] Loewe SMH (1926). über Kombination swirkungen. Arch für Exp Pathol.

[b39] Loewe S (1927). Die Mischarznei. Versuch einer allgemeinen Pharmakologie der Arzneiombinationen. Klin Wochenshrift.

[b40] Loewe S (1953). The problem of synergism and antagonisms of combined drugs. Arzneimittelforsch - Drug Res.

[b41] Loewe S (1959). Randbemerkungen zur quantitativen Pharmakologie der Kombinationen. Arzneimittelforsch - Drug Res.

[b42] Nelson HS (2001). Advair: combination treatment with fluticasone propionate/salmeterol in the treatment of asthma. J Allergy Clin Immunol.

[b43] Nieuwenhuis S, Forstmann BU, Wagenmakers E-J (2011). Erroneous analyses of interactions in neuroscience: a problem of significance. Nat Neurosci.

[b44] Ocana A, Amir E, Yeung C, Seruga B, Tannock IF (2012). How valid are claims for synergy in published clinical studies?. Ann Oncol.

[b45] Oversteegen L, Shah M, Rovini H (2007). HIV combination products. Nat Rev Drug Discov.

[b46] Pangalos MN, Schechter LE, Hurko O (2007). Drug development for CNS disorders: strategies for balancing risk and reducing attrition. Nat Rev Drug Discov.

[b47] Podolsky SH, Greene JA (2011). Combination drugs–hype, harm, and hope. N Engl J Med.

[b48] Prichard MN, Shipman C (1990). A three-dimensional model to analyze drug-drug interactions. Antiviral Res.

[b49] Raffa RB, Stone DJJ, Tallarida RJ (2000). Discovery of “self-synergistic” spinal/supraspinal antinociception produced by acetaminophen (paracetamol). J Pharmacol Exp Ther.

[b50] Shafer SL (2012). All models are wrong. Anesthesiology.

[b51] Shanks N, Greek R, Greek J (2009). Are animal models predictive for humans?. Philos Ethics Humanit Med.

[b52] Slinker BK (1998). The statistics of synergism. J Mol Cell Cardiol.

[b53] Smalley KSM, Haass NK, Brafford PA, Lioni M, Flaherty KT, Herlyn M (2006). Multiple signaling pathways must be targeted to overcome drug resistance in cell lines derived from melanoma metastases. Mol Cancer Ther.

[b54] Straetemans R, O’Brien T, Wouters L, Van Dun J, Janicot M, Bijnens L (2005). Design and analysis of drug combination experiments. Biom J.

[b55] Tallarida RJ (2001). Drug synergism: its detection and applications. J Pharmacol Exp Ther.

[b56] Tallarida RJ (2006). An overview of drug combination analysis with isobolograms. J Pharmacol Exp Ther.

[b57] Tallarida RJ (2012). Revisiting the Isobole and related quantitative methods for assessing drug synergism. J Pharmacol Exp Ther.

[b58] Tsilidis KK, Panagiotou OA, Sena ES, Aretouli E, Evangelou E, Howells DW (2013). Evaluation of excess significance bias in animal studies of neurological diseases. PLoS Biol.

[b59] Wang Y, Zuo J, Wientjes MG (1997). Annual international conference; 22nd, SAS USers Group.

[b60] Whitehead A, Su T-L, Thygesen H, Sperrin M, Harbron C (2013). Investigation of the robustness of two models for assessing synergy in pre-clinical drug combination studies. Pharm Stat.

[b61] Woodcock J, Griffin JP, Behrman RE (2011). Development of novel combination therapies. N Engl J Med.

[b62] Yuan R (2000). Traditional Chinese medicine an approach to scientific proof and clinical validation. Pharmacol Ther.

[b63] Zhao L, Wientjes MG, Au JL-S (2004). Evaluation of combination chemotherapy: integration of nonlinear regression, curve shift, isobologram, and combination index analyses. Clin Cancer Res.

[b64] Zhao L, Au JL-S, Wientjes MG (2010). Comparison of methods for evaluating drug-drug interaction. Front Biosci (Elite Ed).

[b65] Zhao W, Sachsenmeier K, Zhang L, Sult E, Hollingsworth RE, Yang H (2014). A new bliss independence model to analyze drug combination data. J Biomol Screen.

[b66] Zimmermann GR, Lehar J, Keith CT (2007). Multi-target therapeutics: when the whole is greater than the sum of the parts. Drug Discov Today.

