# Corrigendum

**DOI:** 10.1002/prp2.549

**Published:** 2019-11-26

On page 9 of (1), under the section "Issue 6: combining more than two drugs", the following error has been identified in the formula for the Combination Index for the *Bliss Independence* model:

$$\text{CI}=\frac{E_{A}+E_{B}+\ldots +E_{N}-E_{A}E_{B}-E_{A}E_{N}-E_{B}E_{N}-\ldots -E_{A}E_{B}\ldots E_{N}}{E_{AB\ldots N}}$$

The correct formula for the CI for the *Bliss Independence* model in the case of a combination with more than two drugs is:

$$\text{CI}=\frac{\sum_{A\leq i\leq N}E_{i}-\sum_{A\leq i_{1}\leq i_{2}\leq N}E_{i_{1}}E_{i_{2}}+\sum_{A\leq i_{1}\leq i_{2}\leq i_{3}\leq N}E_{i_{1}}E_{i_{2}}E_{i_{3}}+\ldots +{\left(-1\right)}^{N-1}E_{A}E_{B}\ldots E_{N}}{E_{AB\ldots N}}$$

The authors apologize for the error.
