# Supplementary material for: Analysis of drug combinations: current methodological landscape
Source: Pharmacol Res Perspect. 2015 May 20;3(3):e00149. doi: 10.1002/prp2.149 (PMC4492765; doi:10.1002/prp2.149)
Supplement: Supplementary file 1 [file prp20003-e00149-sd1.docx]

**Supplementary material**

**Supplementary 1. Notations.**

Let us consider two drugs A and B in combination.

| $a$ | the dose of drug A administered |
| --- | --- |
| $b$ | the dose of drug B administered |
| $E_{A}$ | the effect of drug A at dose $a$ |
| $E_{B}$ | the effect of drug B at dose $b$ |
| $E_{AB}$ | the effect of the combination of doses $a$ and $b$ |
| $A$ | the single dose of drug A reaching the combination effect $E_{AB}$ |
| $B$ | the single dose of drug B reaching the combination effect $E_{AB}$ |
| $E_{Amax}$ | the maximum effect of drug A |
| $E_{Bmax}$ | the maximum effect of drug B |
| $A_{50}$ | the half maximum effective dose of drug A |
| $B_{50}$ | The half maximum effective dose of drug B |

**Supplementary 2. Dose-effect based strategy: Cases when dose-effect curves are not parallel.**

In practice, only in a limited number of situations are additive isoboles straight lines. The potency ratio ($R$) is often not constant, a situation that would apply when the individual log dose-effect curves are not paralell and/or when the individual drug maximum effects differ and leads to **curvilinear additive isoboles**^1,2^. The calculation of the Combination Index and the isobologram analyses in such situations although more technical, are feasible and described hereafter according to different situations and following the work of Grabovsky and Tallarida (2004)^1^.

Let us consider the drugs A and B of dose-effect curves defined by the Hill equations
$E_{A}= E_{Amax}\times\frac{a^{p}}{A_{50}^{p}+ a^{p}}$ for drug A and $E_{B}= E_{Bmax}\times\frac{b^{q}}{B_{50}^{q}+ b^{q}}$ for drug B. Let us also consider the combination of doses $a$ of drug A and $b$ of drug B producing the effect $E_{AB}$, and the single doses $A$ and $B$ necessary to reach the same effect.

**Situation A: Dose-effect curves with equal maximum (**$\boldsymbol{E}_{\boldsymbol{Amax}}\boldsymbol{=}\boldsymbol{E}_{\boldsymbol{Bmax}}$**) and different shapes (**$\boldsymbol{p \neq q}$**) (Figures A1 and A2).**


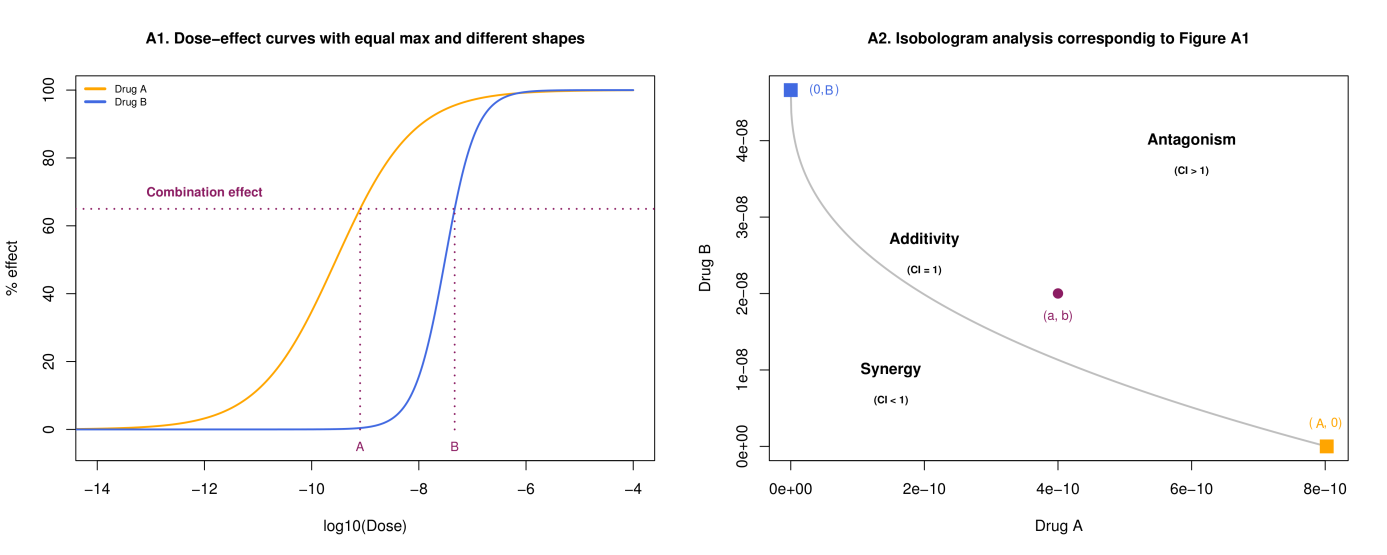


In this situation, the Combination Index is given by:

$$CI= \frac{b}{B}+\frac{B_{50}}{B}\left( \frac{a}{A_{50}} \right)^{\frac{p}{q}}$$

and the equation of the curve of additivity is given by $CI=1$.

**Situation B: Dose-effect curves with different maximum (**$\boldsymbol{E}_{\boldsymbol{Amax}}\boldsymbol{\neq}\boldsymbol{E}_{\boldsymbol{Bmax}}$**) and different shapes (**$\boldsymbol{p \neq q}$**) (Figures B1 and B2).**


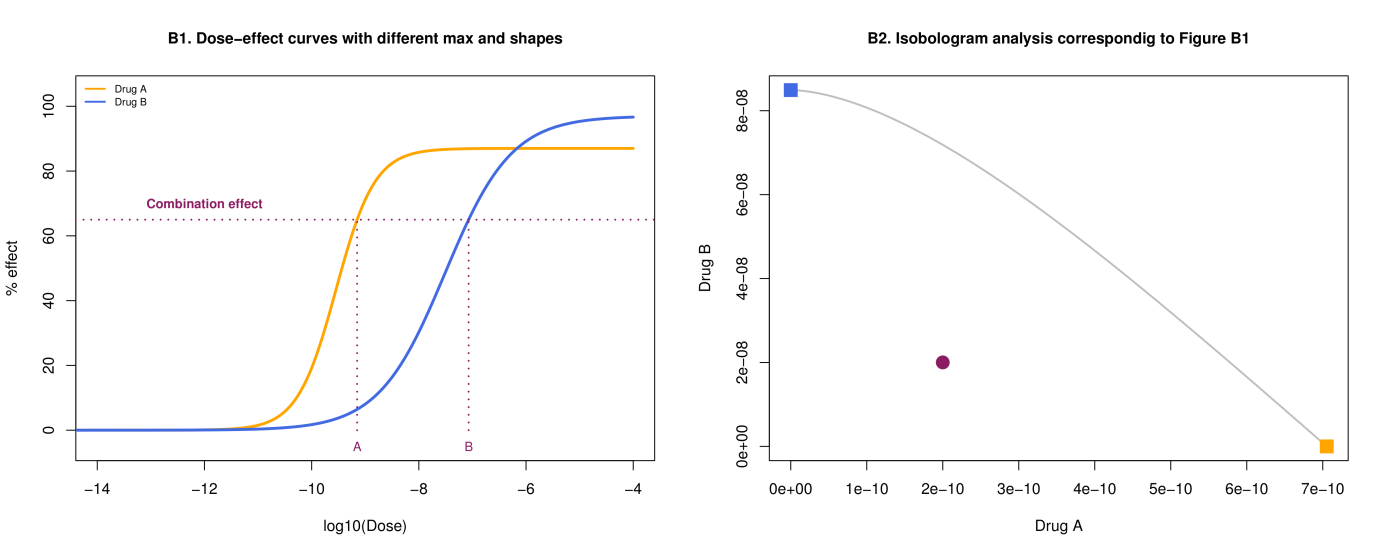


In this situation, the Combination Index is given by:

$$CI= \frac{b}{B}+\frac{B_{50}}{B\left[ \frac{E_{Bmax}}{E_{Amax}}\left( 1+ \frac{{A_{50}}^{p}}{a^{p}} \right)-1 \right]^{\frac{1}{q}}}$$

and the equation of the curve of additivity is given by $CI=1$.

**Situation C: The combination effect is between the maximum individual effects**

**(**$\boldsymbol{E}_{\boldsymbol{Amax}}\boldsymbol{<} \boldsymbol{E}_{\boldsymbol{AB}}\boldsymbol{<}\boldsymbol{E}_{\boldsymbol{Bmax}}$ **for instance) (Figures C1 and C2).**


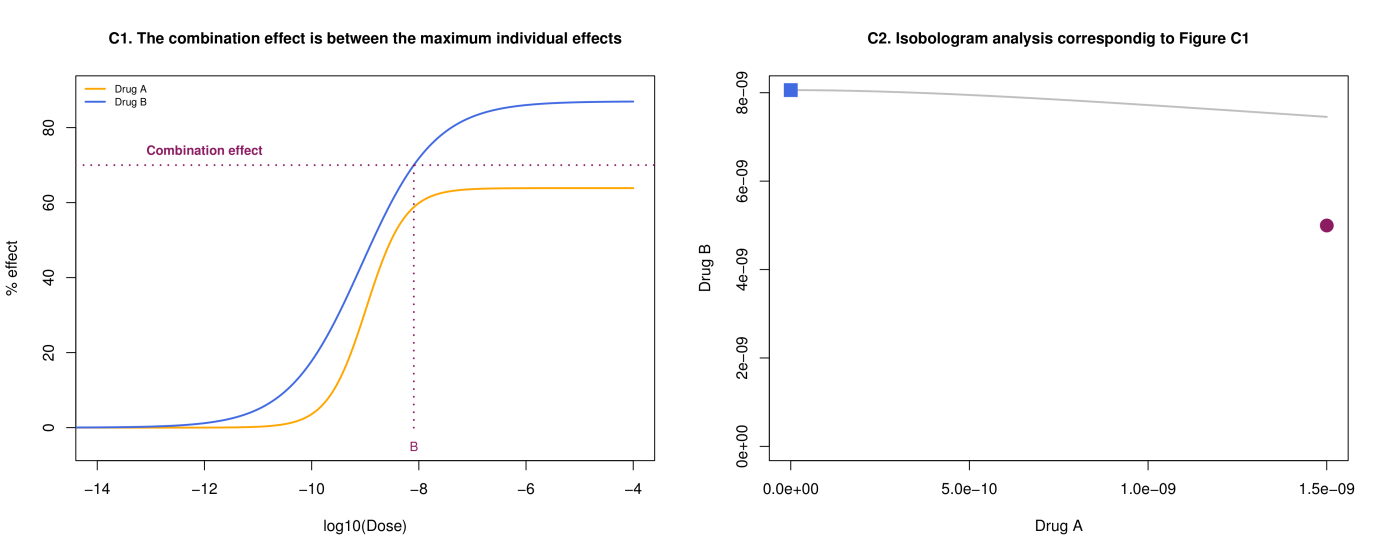


In this situation, the Combination Index is given by:

$$CI= \frac{b}{B}+ \frac{1}{B}\left( \frac{1}{\frac{1}{B_{A}^{q}}+\frac{\gamma}{a^{p}}} \right)^{\frac{1}{q}}$$

where $B_{A}$ is the dose of drug B that produces the maximum effect of drug A and $\gamma=\left( E_{Bmax}{A_{50}}^{p} \right)/\left( E_{Amax}{B_{50}}^{q} \right)$.

**Situation D: The combination effect is greater than the maximum effect of both drugs**

**(**$\boldsymbol{E}_{\boldsymbol{AB}}\boldsymbol{>}{\boldsymbol{E}_{\boldsymbol{Amax}} \mathbf{and} \boldsymbol{E}}_{\boldsymbol{Bmax}}$**) (Figure D).**

**
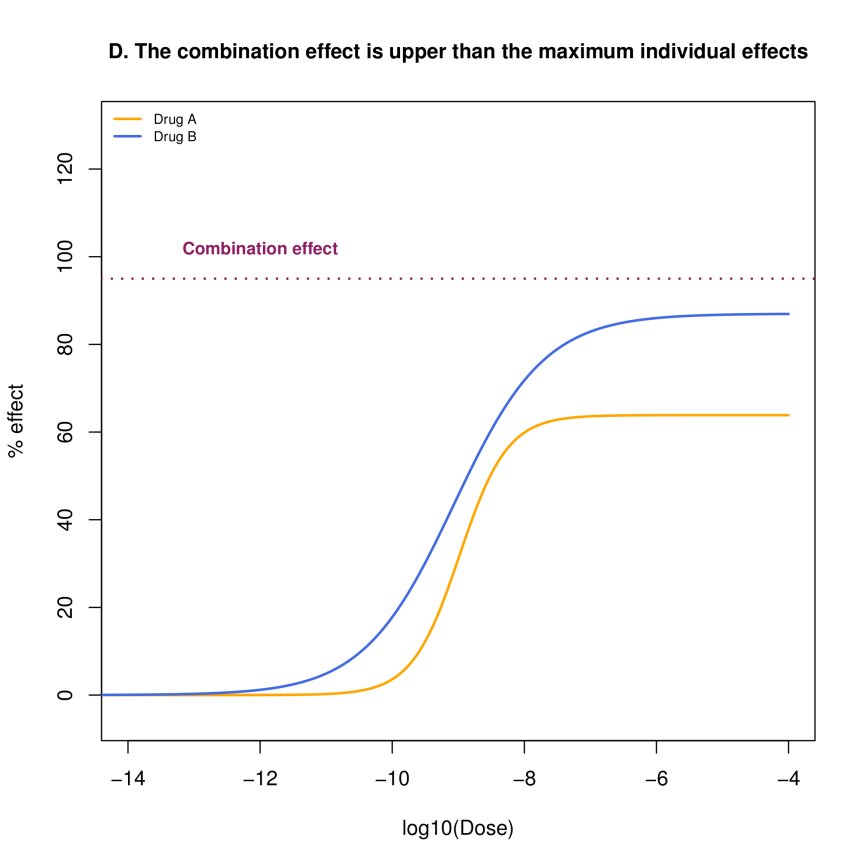
**

In this situation, a Combination Index cannot be calculated properly but a ‘boost’ *P*-value can be calculating by comparing the combination effect to the maximum individual effect and can be interpreted as absolute synergism translated by an increase in maximum efficacy in case of significance.

**References**

1. Grabovsky, Y. & Tallarida, R. J. Isobolographic analysis for combinations of a full and partial agonist: Curved isoboles. *J. Pharmacol. Exp. Ther.* **310,** 981–986 (2004).

2. Geary, N. Understanding synergy. *Am. J. Physiol. Metab.* **304,** E237–E253 (2013).
